# Curcumin Induces Transgenerational and Sex‐Specific Effects on Lifespan, Gene Expression, and Metabolism in the Fruit Fly *Drosophila melanogaster*

**DOI:** 10.1002/biof.70039

**Published:** 2025-08-01

**Authors:** Silvana Hof‐Michel, Belén Olga Ferrando Hernandez, Andreas Vilcinskas, Anika E. Wagner

**Affiliations:** ^1^ Institute of Nutritional Science Justus Liebig University Giessen Germany; ^2^ Department of Food Technology, Food Sciences and Nutrition, Campus of International Excellence “Campus Mare Nostrum” University of Murcia Murcia Spain; ^3^ Branch Bioresources Fraunhofer Institute for Molecular Biology and Applied Ecology IME Giessen Germany; ^4^ Institute for Insect Biotechnology Justus Liebig University Giessen Germany; ^5^ Center for Sustainable Food Systems Justus Liebig University Giessen Germany

**Keywords:** aging, curcumin, *Drosophila melanogaster*, epigenetics, nutrition, sex‐specific, transgenerational

## Abstract

Curcumin is a bioactive compound found in turmeric ( *Curcuma longa* ) and is widely recognized for its health‐promoting effects, including anti‐inflammatory, antioxidant, and anti‐carcinogenic properties. It can also mediate epigenetic effects by inhibiting histone acetylases (HATs) and deacetylases (HDACs) but the transgenerational context has not been studied in detail. Here, we used the fruit fly ( *Drosophila melanogaster* ) as a model organism to determine the epigenetic effects of 0.1% and 1% (w/v) curcumin, which have been shown to promote the health and prolong the lifespan of fruit flies. Both concentrations were found to significantly increase lifespan and climbing activity in male and female flies, but changes in HAT/HDAC gene expression and metabolism were sex‐specific. Unexpectedly, the F1 offspring of curcumin‐treated parental flies showed a significant reduction in lifespan that was also sex‐specific, as well as sex‐specific and dose‐dependent transgenerational changes in HAT/HDAC gene expression and metabolism. These results show that curcumin's beneficial effects in the parental generation are followed by deleterious effects in the offspring, highlighting the need to further investigate the potential transgenerational effects of nutrients and bioactive compounds that are used as dietary supplements for humans.

## Graphical abstract

Curcumin feeding prolongs lifespan in male and female flies, while their offspring showed a reduced lifespan in a sex‐dependent manner. Climbing activity, a measure of fitness, is also enhanced in the F0, but not in the F1, whereas gene expression levels of epigenetic markers are affected in both generations.

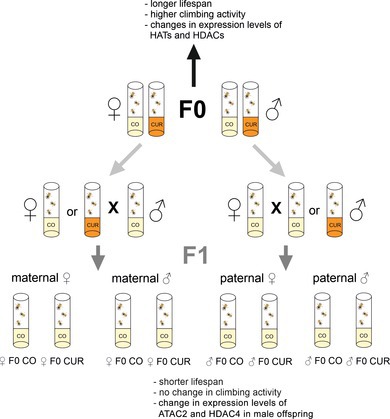

**Abbreviations**
ATAC2: Ada2a‐containing complex component 2
CUR: curcumin
Enok: enoki mushroom
F0: parental generation
F1: first filial generation
HAT: histone acetyltransferase
HAT1: histone acetyltransferase 1a
HDAC: histone deacetylase
HDAC4: histone deacetylase 4
HDAC6: histone deacetylase 6
ISCs: intestinal stem cells
NF‐κB: nuclear factor “kappa‐light‐chain‐enhancer” of activated B‐cells
PBS: phosphate buffered saline
Rpd3: reduced potassium dependency 3
Rpl32: ribosomal protein L32
Rps20: ribosomal protein S20
Sirt2: Sirtuin 2
TAG: triacylglycerides
Tip60: Tat interactive protein 60 kDa

## 1 Introduction

Curcumin, a polyphenolic compound derived from the rhizomes of turmeric ( *Curcuma longa* ), has been used for more than 1000 years as a spice, food coloring, and wound healing remedy [1, 2, 3, 4, 5]. It is known for its anti‐inflammatory and antioxidant properties, making it a compound of interest in cancer therapy [6, 7]. One notable mechanism involves its inhibition of histone acetyltransferases (HATs) and histone deacetylases (HDACs) which facilitate the epigenetic regulation of gene expression [8] without altering the underlying DNA sequence [9]. HATs add acetyl groups to histone proteins in chromatin, resulting in a more open chromatin structure that facilitates transcription, whereas HDACs remove these acetyl groups, leading to a more condensed chromatin structure that is less accessible for transcription factors and RNA polymerases [8]. Histone acetylation/deacetylation regulates transcriptional reprogramming in insects during metamorphosis, wounding, and infection [10]. The ability of curcumin to inhibit HATs and HDACs suggests it plays a complex role in epigenetic processes.

Dietary components are known to elicit epigenetic effects that affect complex traits such as longevity [11]. Transgenerational epigenetic effects induced by diet were first experimentally shown in rats [12], but evidence for such changes in humans has been shown in individuals born during the Dutch Hunger Winter in 1944–1945. The exposure of pregnant women to malnutrition and famine leads to metabolic syndrome phenotypes and altered DNA methylation patterns in their children [13]. Transgenerational experiments in humans and other mammals are complex and subject to strict ethical restrictions, so invertebrate models, such as the fruit fly *Drosophila melanogaster* , are often used instead [14, 15, 16, 17]. Fruit flies offer several advantages for such experiments, including a short generation time, ease of handling, low cost, and ~75% identity with human genes [18, 19]. Importantly, the epigenetic mechanisms and orthologs of key genes involved in human health are also present in flies [20]. Several studies have shown that dietary interventions in the fruit fly are relatively easy [21, 22]. The combination of a short generation time, homologous genes and mechanisms, and the ease of dietary manipulation makes the fruit fly highly suitable to study the transgenerational epigenetic effect of food supplements [23].

Previous reports have demonstrated the direct impact of curcumin on lifespan and health, particularly in fruit flies [24, 25, 26, 27]. However, the transgenerational impact of curcumin is only rarely considered. Curcumin toxicity testing in rats showed that the highest parental dose caused a low body weight in the F2 generation [28]. The treatment of fruit flies with low concentrations of curcumin (5–10 μM) was shown to increase the viability of F1 offspring [29]. Transgenerational effects of curcumin on longevity and gene expression were also shown in the red flour beetle *Tribolium castaneum* [30].

Here we investigated the transgenerational effect of curcumin in fruit flies, focusing on the potentially detrimental impact of higher doses [7, 31, 32, 33, 34] and sex‐specific effects on lifespan and health [35, 36, 37]. Offspring were only reared on food without curcumin and were never exposed to that compound, allowing us to isolate transgenerational effects from acute exposure. We report that curcumin at still beneficial concentrations for parental flies has a clear transgenerational impact on lifespan, metabolism, and gene expression in the F1 generation, including a reduced lifespan and changes in the expression profiles of selected HAT and HDAC genes, in a sex‐specific manner, showing a clear difference between maternal and paternal treatments. This highlights the need for sex‐specific considerations in experimental design and emphasizes the need for further research into the transgenerational effects of curcumin in higher organisms, especially in humans, where curcumin is widely used as a health‐promoting ingredient in foods.

## 2 Experimental Procedures

### 2.1 Maintenance of Flies

All experiments were conducted using the *D. melanogaster* mutant strain w^1118^ (Bloomington Drosophila Stock Center, IN, USA; #5905) reared at 25°C and 60% humidity with a 12‐h photoperiod in a climate chamber (Memmert, Schwabach, Germany) and were fed on CT medium, as previously described [38]. The experimental diet (SY 10%) consisted of 10% sucrose (Carl Roth, Karlsruhe, Germany), 10% inactive dry yeast (Genesee via Kisker, Steinfurt, Germany), 2% agar (Apex via Kisker), 0.3% propionic acid (Carl Roth), and 1.5% tegosept (Apex via Kisker) [39]. For nutritional intervention, SY 10% was supplemented with either 1% or 0.1% (w/v) curcumin (Santa Cruz Biotechnology, Dallas, TX, USA; SC‐200509), and stirred at 60°C for 20–25 min. SY 10% without curcumin was used as the control diet.

### 2.2 Age‐Matched Flies and Lifespan Experiments

Age‐matched flies were prepared as previously described [39]. Briefly, 300–400 male and female flies were transferred to embryo collection cages (Genesee via Kisker) and sealed with grape juice agar (Genesee via Kisker) plates containing active yeast paste (Genesee via Kisker). After 24 h for adaptation, the plates were replaced, and eggs were collected overnight. The next day, flies were discarded, and eggs were washed from the agar plates with phosphate buffered saline (PBS, pH 7.4; Thermo Fisher Scientific, Schwerte, Germany) using a cotton swab. The eggs were then washed 2–3 times with PBS to remove residual yeast paste. We then transferred 35 μL of eggs to bottles containing CT medium and incubated them in the climate chamber until they hatched. After hatching, flies were allowed to mate for 48 h. For lifespan analysis, flies were separated by sex, and 25 flies of each sex were allocated to vials containing either the curcumin‐supplemented or control diets. Flies were transferred to fresh food vials every 2–3 days, and the number of dead flies per vial was recorded until all flies were dead. Three independent experiments were carried out, each with three vials per sex and treatment. Data were pooled for statistical analysis.

### 2.3 Generation of F1 Flies

To investigate the effect of parental diets on F1 offspring, female (maternal effect) or male (paternal effect) flies were fed with curcumin. For maternal treatment, male and female flies were separated 2 days after hatching. To avoid fertilization by untreated males, virgin females were collected for the paternal treatment. Female (maternal) or male (paternal) flies were fed on diets supplemented with 0.1% or 1% curcumin or the control diet for 7 days. The corresponding partner flies (males for the maternal treatment and females for the paternal treatment) were fed on the control diet. For oviposition, male and female flies were placed together again in bottles containing CT medium for optimal larval development. Parental flies were discarded after 24 h. After hatching, male and female offspring were kept together for 2 days to allow mating. Flies were then separated by sex for further experiments and maintained on the control diet.

### 2.4 Gustatory Assay

To determine food intake, 23 age‐matched male and female flies per vial were fed on diets either supplemented with 0.1% or 1% curcumin or the control diet for 5 days and were transferred to fresh food vials on day 3. Flies were then placed in vials containing the same diet supplemented with 0.2% of sulforhodamine B sodium salt (Sigma‐Aldrich, MO, USA). After 8 h, flies were frozen at −80°C overnight. Twenty flies per sample were then homogenized in 200 μL PBS (pH 7.4) containing 1% Triton X‐100 (Sigma‐Aldrich) using a Tissue Lyser II (Qiagen, Hilden, Germany) at a frequency of 25 s^−1^ for 6 min. Samples were centrifuged (4000 × g, 5 min, 4°C) and transferred to Eppendorf tubes. Fluorescence was measured in triplicate on a SpectraMax iD3 microplate reader (Molecular Devices, San Jose, CA, USA) at an excitation wavelength of 535 nm and an emission wavelength of 590 nm. Flies fed with unstained food were used for background correction. Fluorescence values were calculated from a standard curve based on serial dilutions of sulforhodamine B sodium salt.

### 2.5 Climbing Assay

To determine climbing ability, age‐matched flies were separated by sex (30 flies per vial) and maintained on curcumin‐supplemented or control diets. After 10, 20, and 30 days, flies were transferred to empty vials with marks at 3 and 6 cm height, and were placed inside a custom‐made climbing bracket (Labortechnik von Keutz, Reiskirchen, Germany). Flies were allowed to recover from the transfer for 30 min. The bracket was then placed in front of an EOS M50 camera (Canon, 's‐Hertogenbosch, Netherlands) for 5 min before recording. After recording was started, flies were tapped down three times at 90s intervals. Flies that were 10 or 20 days old were transferred back to fresh food‐vials whereas those aged 30 days were weighed and frozen at −80°C for further analysis. The climbing score was calculated as the sum of the percentage of flies in each section of the vial 10 s after tapping multiplied by the assigned section number (see Figure S1). The mean of the repeated taps was used as the final climbing score to equalize for differences in tapping.

### 2.6 Activity Assay

To analyze the activity pattern, age‐matched flies were sorted by sex (20 flies per vial) and continuously fed with the curcumin‐supplemented or control diet. Vials were placed into a LAM25 Locomotor Activity Monitor (TriKinetics, Princeton, NJ, USA) in the climate chamber. Vials were changed every 2–3 days, and the number of dead flies was counted. The activity monitor was connected to a computer for data collection using the DAMSystem312X software. Flies crossing the infrared beam were summarized at 10 min intervals. The data were separated into four light phases (day, dusk, night and dawn), where day is 100% light (6500 K), dusk 90%–10% light, night 0% light, and dawn 10%–90% light. Data were visualized for graphic representation using ActogramJ in FIJI [40] and locomotor activity in counts per time period was statistically analyzed using SPSS Statistics, v26.0.0.1 for Windows (IBM, Armonk, NY, USA).

### 2.7 Metabolic Composition

For the analysis of glucose, protein, and triacylglyceride (TAG) levels, flies (30 flies per vial) were fed on the curcumin‐supplemented or control diets for 8 or 30 days. On day 8, flies were frozen at −80°C overnight without weighing. On day 30, flies were weighed and then frozen at −80°C overnight. Five flies per sample were homogenized in 250 μL PBS containing 1% Triton X‐100 using a Tissue Lyser II as above. Homogenates were centrifuged (5000 × g, 10 min, 4°C) and kept at room temperature for 30 min. The debris‐free supernatants were transferred to fresh Eppendorf tubes and assessed for protein using a Roti‐Quant universal assay (Carl Roth), for TAGs using a GPO‐PAP assay, and for glucose using a GOD‐PAP assay (both Dialab, Neudorf, Austria) according to the manufacturers' instructions. Before glucose measurements, samples were heated to 70°C for 5 min and cooled to room temperature for 20 min, and the centrifugation step was repeated.

### 2.8 RNA Isolation and Real‐Time RT‐PCR

RNA was isolated using the Quick‐RNA Tissue/Insect kit (Zymo Research, Freiburg, Germany) according to the manufacturer's instructions. Briefly, 10 flies were homogenized in RNA lysis buffer and transferred to column tubes for RNA binding and washing. Residual DNA was eliminated by incubating with DNase I (Zymo Research). RNA was eluted into DNase/RNase‐free water, and the concentration was determined at 230/260/280 nm using a UVmini‐1240 UV–VIS spectrophotometer (Shimadzu, Duisburg, Germany). RNA was stored at −80°C. For cDNA synthesis, 3 μg of RNA was diluted in 34.5 μL DNase/RNase‐free water and mixed with 3 μL of the oligo(dT) primer (20 ng/μL; Promega, Mannheim, Germany), 12 μL of MLV RT 5× Buffer (Promega), 1.5 μL of RNasin RiboLock (40 U/μL; Sigma‐Aldrich), 6 μL of dNTP mix (10 mM; Promega), and 3 μL of M‐MLV reverse transcriptase (200 U/μL; Promega). Samples were incubated at 42°C for 60 min in a T‐Gradient Thermo Block (Biometra, Göttingen, Germany) and then heated to 70°C for 10 min to inactivate the enzyme. The cDNA was stored at −80°C. For real‐time PCR, 2 μL of cDNA per well in a 96‐well plate was mixed with 18 μL of master mix containing 10 μL PerfeCTa SYBR Green SuperMix, Low ROX (Quantabio, Beverly, MA, USA), 0.25 μL of each of the forward and reverse primers (10 pmol/μL; Eurofins Genomics, Ebersberg, Germany) and 7.5 μL DNase/RNase‐free water. Semi‐quantitative real‐time PCR was carried out using a 7500 Real Time PCR system (Applied Biosystems, Heidelberg, Germany). We measured transcript levels for the HAT/HDAC genes *ATAC2*, *enok*, *Tip60*, *HAT1*, *Rpd3*, *Sirt2*, *HDAC4*, and *HDAC6*. *RpL32* and *RpS20* were used as reference genes. All primer sequences are listed in Table 1.

**TABLE 1:** Primer sequences for selected HAT and HDAC genes.

Gene	Primer forward (5′ → 3′)	Primer reverse (3′ → 5′)	Temp.
*RpL32*	GGCAAGCTTCAAGATGACCA	GTTCGATCCGTAACCGATGT	55°C
*RpS20*	TGTGGTGAGGGTTCCAAGAC	GACGATCTCAGAGGGCGAGT	58°C
*ATAC2*	ATCTCAGTGGCGGATCATGC	CGGCCCATGTAGGGAATTGT	55°C
*enok*	CGGCCAAGCAAAAAGTCGAA	CCGCCTCCCACTTGAAGATT	55°C
*Tip60*	GTCCAAGTTCGAGGGCAAGA	TGCATTCGCAGATGTCGTTTA	55°C
*HAT1*	GTGGTGTTACTTCTTGAGCTACG	GGTAGCCAAGCCGAGTTTCT	58°C
*Rpd3*	GAAAAGGAAAAGGGCGACGG	TCGCTCCGGCATCATTATCG	58°C
*Sirt2*	TGGGCGACATCGAAGTAAAG	TATCCATTGTGTCGTCTGGG	56°C
*HDAC4*	CGAATGGCACAACGACAACG	GTTCGGTTCGGATGCTGTCT	57°C
*HDAC6*	AGCTGTGAATCGCTCCGTCC	GATGGGCGGGCTCATGTTT	57°C

Abbreviations: ATAC2, Ada2a‐containing complex component 2; enok, enoki mushroom; HAT1, Histone acetyltransferase 1a; HDAC4, histone deacetylase 4; HDAC6, histone deacetylase 6; Rpd3, Reduced potassium dependency 3; Rpl32, Ribosomal protein L32; Rps20, Ribosomal protein S20; Sirt2, Sirtuin2; Tip60, Tat interactive protein 60 kDa.

### 2.9 Statistical Analysis

All experiments were carried out in triplicate. Data were normalized to the mean of the time‐matched control before further analysis. Residuals were tested for normality of distribution (Shapiro–Wilk) and homogeneity of variances (Levene's test based on the mean). Following these tests, we applied ordinary one‐way analysis of variance (ANOVA), Welch‐ANOVA (Games‐Howell correction) or Kruskal‐Wallis test, as appropriate. For interaction between sexes, we used two‐way ANOVA or two‐way Kruskal–Wallis with group × sex as the test of between‐subjects' effects, as previously described [41, 42]. We used mixed model analysis with a random intercept model for climbing assay and activity assay data, allowing for the dependencies in the model. Kaplan–Meier statistics followed by a log‐rank test were used to analyze survival data. If not stated otherwise, multiple comparisons were corrected using the Bonferroni‐Dunn method. Significance was accepted at *p* < 0.05. GraphPad Prism v10.4 for Windows (GraphPad Software, Boston, MA, USA) was used for lifespan analysis and graph presentation, whereas SPSS Statistics v26.0.0.1 for Windows was used for all other statistical tests. CorelDRAW v24.2.2.446, 2022 was used to create illustrations.

## 3 Results

### 3.1 Curcumin Supplementation Affects the Food Intake and Lifespan of F0 Flies

**FIGURE 1:**
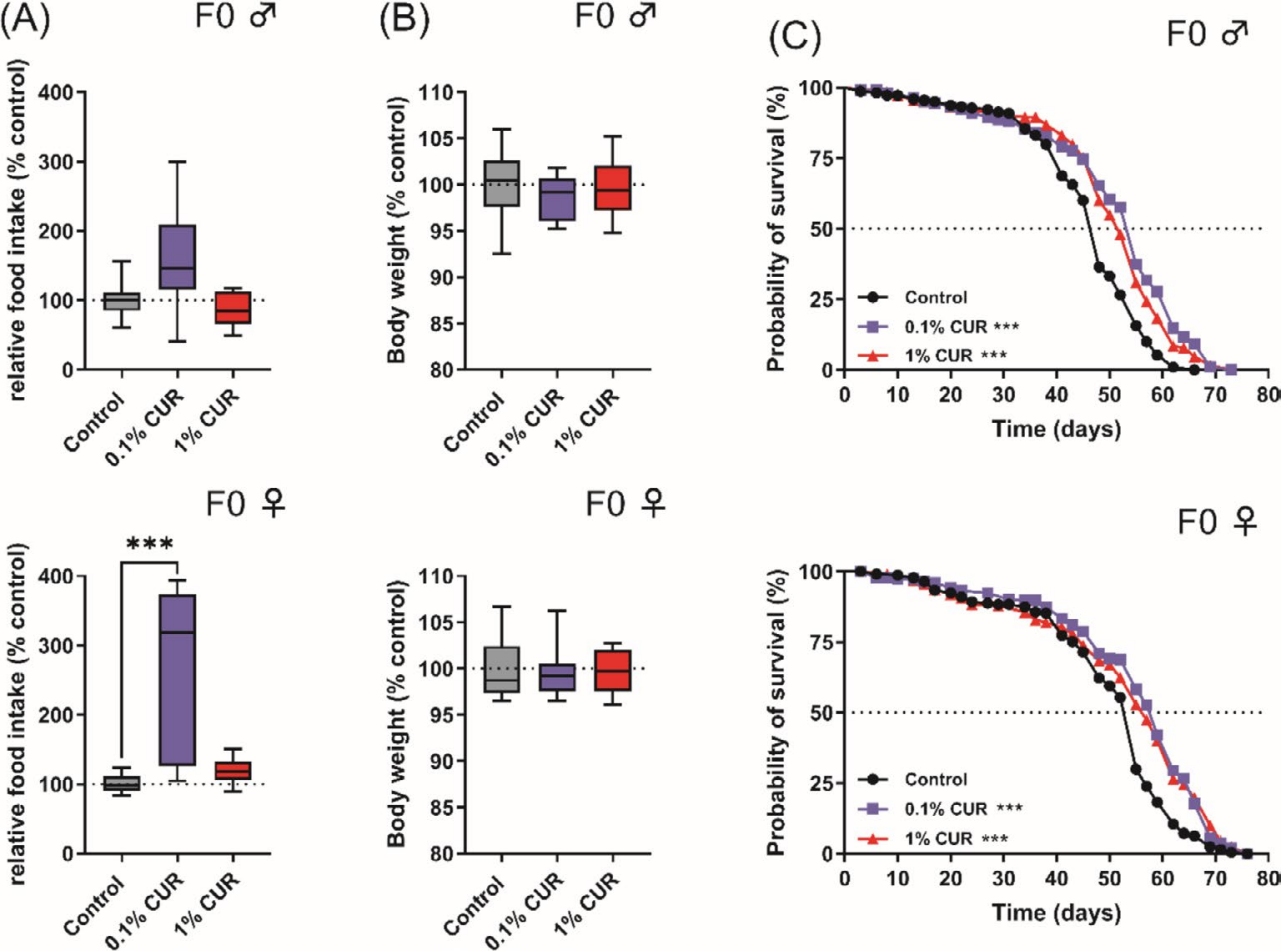
Curcumin increases food intake and enhances median lifespan in fruit flies. Food intake and body weight were measured after 5 days of treatment. (A) Food intake was not significantly changed by curcumin treatment in male flies, but female flies treated with 0.1% curcumin showed an increased food intake. (B) No significant changes in body weight were observed after 5 days. (C) Treatment with curcumin increased median lifespan in male and female flies. Boxplots in (A) and (B) show median, minimum, and maximum values. Lifespan is represented as Kaplan–Meier curves. Data represent three independent experiments, each with three replicates. (A) Kruskal–Wallis (B) ANOVA or Kruskal‐Wallis (C) Log‐rank Test (***p < 0.001). CUR, curcumin.

We analyzed the food intake of parental flies to exclude the possibility that curcumin causes caloric restriction. Male and female flies were separated after mating and fed on diets supplemented with 0.1% or 1% curcumin or the control diet for 5 days. The analysis of relative food intake and body weight in male flies revealed no significant differences. However, female flies on the 0.1% curcumin diet showed a 175% increase in food intake compared to the control diet, but no significant difference in body weight (Figure 1A,B), even after 30 days (Figure S2). Lifespan analysis demonstrated a significant increase in the median lifespan of male and female flies on either of the supplemented diets, with 0.1% curcumin achieving a more pronounced effect (Figure 1C; ♂ 0.1% curcumin +14.6%, 1% curcumin +8.4%; ♀ 0.1% curcumin +7.3%, 1% curcumin +3.6%). Furthermore, the mean and maximum lifespan (final 10% of living flies) were also significantly higher in males on the 0.1% curcumin diet, but there was no corresponding effect in females (Table 2).

**TABLE 2:** Median, mean, and maximum lifespan of F0 flies.

Treatment	Median lifespan		Mean lifespan		Maximum lifespan	
♂ Control	48 days	100%	100%		100%	
♂ 0.1% CUR	55 days	114.6%***	111% ± 2.3%	**	112% ± 3.4%	*
♂ 1% CUR	52 days	108.4%***	110% ± 4.2%	n.s.	110% ± 3.4%	n.s.
♀ Control	55 days	100%	100%		100%	
♀ 0.1% CUR	59 days	107.3%***	111% ± 14.2%	n.s.	105% ± 4.5%	n.s.
♀ 1% CUR	57 days	103.6%***	106% ± 11.7%	n.s.	102% ± 6.3%	n.s.

*Note:* n.s., not significant. Presented are median lifespan with log‐rank test and mean and maximum lifespan (last 10% of living flies) with ANOVA in mean ± SD, respectively. CUR, curcumin.

* *p* < 0.05.

** *p* < 0.01.

*** *p* < 0.001.

### 3.2 Curcumin Increases the Climbing Ability of F0 Flies but Decreases Activity Levels

**FIGURE 2:**
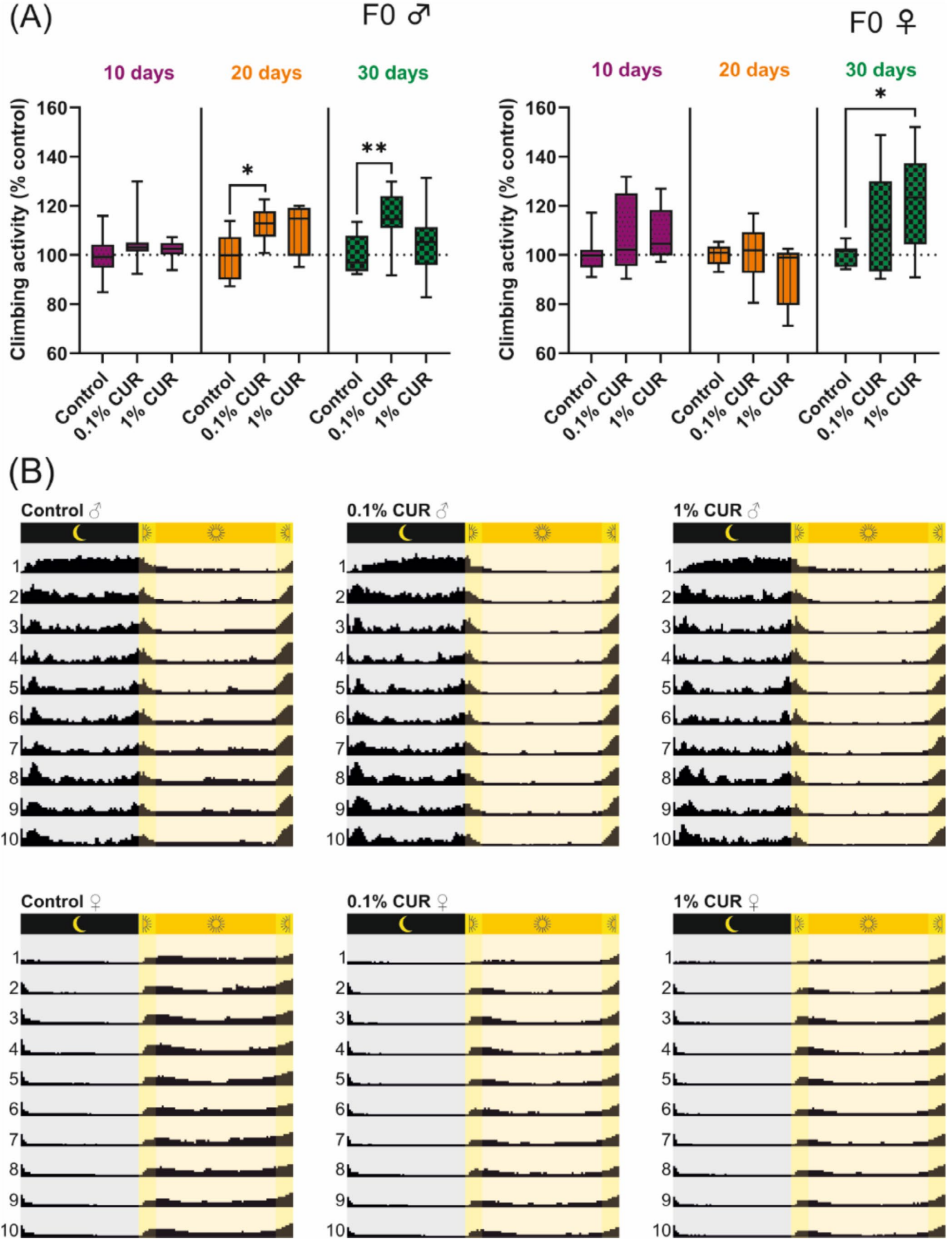
Curcumin increases the climbing ability of flies while reducing their overall activity. (A) Climbing ability is enhanced in male flies after 20 days of treatment with 0.1% curcumin and is still visible after 30 days. Female flies show enhanced climbing ability after 30 days of treatment with 1% curcumin. (B) Graphic representation of activity levels, which differ between male and female flies and are reduced by curcumin treatment in both sexes. Activity values are listed in Table 3. The boxplots in panel A show median, minimum, and maximum values. Data represent three independent experiments, each with three replicates. (A) Mixed model analysis (*p > 0.05; **p > 0.01). CUR, curcumin.

The climbing assay assesses the natural climbing behavior of flies as a fitness parameter. Climbing behavior has been found to decline naturally with age but is also influenced by nutritional status [43]. The climbing assay was carried out after 10, 20, and 30 days of treatment to assess any differences caused by aging. Male flies on the 0.1% curcumin diet showed a 12% increase in climbing activity after 20 days, rising to 15% after 30 days. In contrast, female flies on the 1% curcumin diet showed an 8% increase in climbing ability after 10 days, followed by a drop to 8% below the baseline after 20 days and then a significant increase to 23% above the baseline after 30 days (Figure 2A). Male flies on the 1% curcumin diet and female flies on the 0.1% curcumin diet did not show significant differences in climbing activity. To investigate these behavioral patterns in more detail, we measured the activity levels of flies continuously over a period of 10 days. Monitoring revealed clear differences in activity within the 24‐h diurnal cycle (day, dusk, night and dawn) and between sexes (Figure 2B). Male flies showed no significant variation in activity levels between dusk and dawn (*p* = 0.114) or day and night (*p* = 0.566), whereas female flies showed differences between dusk and dawn (*p* = 0.034) and day and night (*p* = 0.018). In general, male and female flies on the control diet showed differences in activity levels during the night (*p* < 0.0001) and dusk (*p* = 0.001), with male flies showing higher activity (dusk, ♂ 1656 vs. ♀ 310; night, ♂ 7232 vs. ♀ 2531). Curcumin reduced activity levels during dawn (control 497, 0.1% curcumin 147, 1% curcumin 195) and day (control 6369, 0.1% curcumin 3951, 1% curcumin 3881) in male flies, whereas female flies fed on 1% curcumin were affected at all time points. Feeding on 0.1% curcumin also reduced activity levels in female flies, with significant differences during dusk, night, and day. All activity levels are summarized in Table 3.

**TABLE 3:** Activity levels of curcumin‐treated flies in the F0 generation.

Treatment	Dusk	Night	Dawn	Day
♂ Control	1656 ± 973	7232 ± 3802	497 ± 228	6369 ± 1570
♂ 0.1% CUR	1595 ± 783	6936 ± 3127	**147 ± 109** ***p* = 0.002** **	**3951 ± 971** ***p* = 0.002** **
♂ 1% CUR	1515 ± 635	6366 ± 3091	**195 ± 163** ***p* = 0.006** **	**3881 ± 984** ***p* = 0.002** **
♀ Control	310 ± 173	2531 ± 789	1119 ± 410	6742 ± 1730
♀ 0.1% CUR	**203 ± 107** ***p* = 0.01** **	**1835 ± 596** ***p* = 0.038** *	690 ± 235	**3865 ± 1119** ***p* = 0.022** *
♀ 1% CUR	**159 ± 83** ***p* < 0.001** ***	**1591 ± 562** ***p* = 0.01** **	**605 ± 239** ***p* = 0.038** *	**3372 ± 1067** ***p* = 0.012** *

*Note:* Comparison to same sex control with mixed model analysis. Presented are the mean activity counts over 10 days ±SD. CUR, curcumin.

* *p* < 0.05.

** *p* < 0.01.

*** *p* < 0.001.

### 3.3 Impact of Curcumin on Metabolic Changes During Aging

**FIGURE 3:**
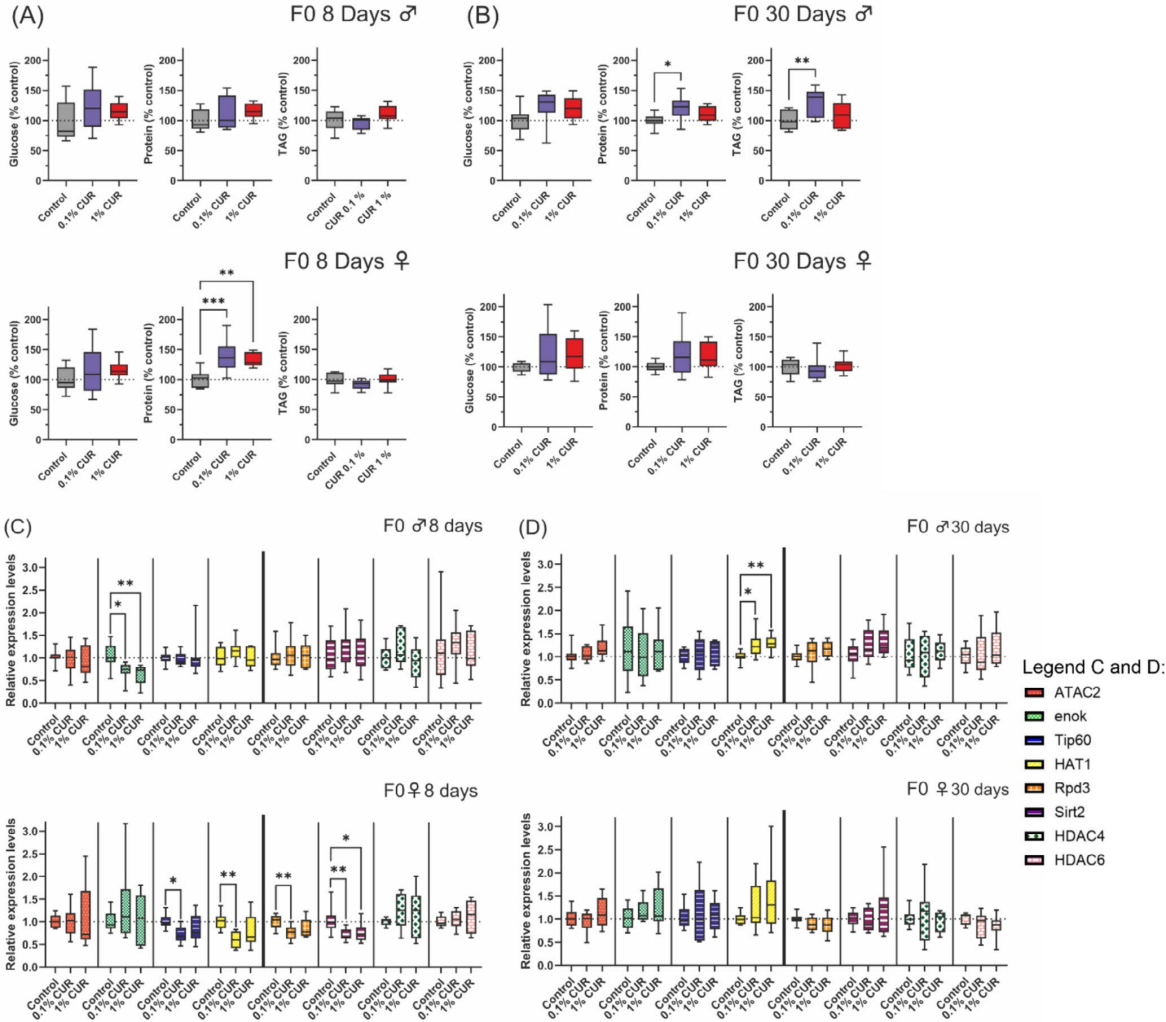
Changes in metabolism and gene expression after curcumin treatment after 8 and 30 days of treatment. Total levels of glucose, protein, and triacylglycerol (TAG), and the expression levels were analyzed after 8 and 30 days of treatment. (A) Total levels of glucose, protein, and TAG after 8 days show increased protein levels in female flies. (B) Total levels of glucose, protein, and TAG after 30 days show increased protein and TAG levels in male flies fed on 0.1% curcumin. (C) Expression levels of selected HAT and HDAC genes after 8 days show a reduction of *enok* expression in male flies and a reduction of *Tip60*, *HAT1*, *Rpd3*, and *Sirt2* expression in female flies fed on 0.1% curcumin, as well as lower *Sirt2* expression in flies fed on 1% curcumin. (D) Expression levels of selected HAT and HDAC genes after 30 days show increased levels of *HAT1* expression in male flies. Boxplots show median, minimum, and maximum values. Data represent three independent experiments, each with three replicates. (A) ANOVA (B) ANOVA or Welch‐ANOVA (C and D) ANOVA or Welch‐ANOVA or Kruskal–Wallis (*p > 0.05; **p < 0.01; ***p < 0.001). CUR, curcumin.

Metabolic changes are caused not only by nutritional factors but also by the process of aging itself, so we analyzed levels of glucose, proteins, and TAGs after 8 and 30 days of treatment. Eight days was selected to observe possible metabolic differences between treated and untreated flies at the time when the offspring (F1) would be produced by the F0 parents, and 30 days was selected to observe changes that occur during aging. No alterations in glucose levels were detected at any time point, whereas protein levels in female flies increased significantly by 38% (0.1% curcumin) and 33% (1% curcumin) after 8 days but returned to control levels after 30 days. In contrast, male flies showed elevated protein levels after 30 days on the 0.1% curcumin diet. TAG levels fell slightly in both sexes on the 0.1% curcumin diet (by 5% in males and 8% in females, in both cases not statistically significant due to high standard deviations). In contrast, male flies on the 0.1% curcumin diet for 30 days showed a significant 30% increase in TAG (Figure 3A,B).

### 3.4 HAT and HDAC Gene Expression Are Affected by Curcumin Treatment

Given that curcumin inhibits HAT and HDAC gene expression, we selected the HAT genes *ATAC2*, *enok*, *Tip60*, and *HAT1*, as well as the HDAC genes *Rpd3*, *Sirt2*, *HDAC4*, and *HDAC6* (representing each HDAC class in flies) for expression analysis in flies fed on curcumin‐supplemented diets for 8 and 30 days. After 8 days, the only affected gene in male flies was *enok*, with −30% and −39% lower expression levels in flies on the 0.1% and 1% curcumin diets, respectively. In contrast, the *Tip60*, *HAT1*, *Rpd3*, and *Sirt2* genes were affected in female flies at the same time point, with the expression level of all four genes reduced in flies on the 0.1% curcumin diet, and *Sirt2* expression also downregulated in flies on the 1% curcumin diet (Figure 3C). After 30 days, *enok* expression in males had returned to control levels, but the expression of *HAT1* had increased by 25% and 29% on the 0.1% and 1% curcumin diets, respectively. In females, the expression levels of *Tip60*, *HAT1*, *Rpd3*, and *Sirt2* hat returned to control levels after 30 days (Figure 3D).

### 3.5 Curcumin Induces Sex‐Dependent Transgenerational Effects

Having assessed the effect of curcumin in parental flies (F0), we turned our attention to the effect in F1 flies on a control diet following either maternal or paternal exposure to curcumin. Synchronized flies were prepared as described above, with virgin females collected and maintained on the control diet for 1 week for paternal treatment. Corresponding male flies were fed on diets supplemented with either 0.1% curcumin, 1% curcumin, or the control diet. On day 8 of treatment, male and female flies were placed together again for egg‐laying. For the maternal treatment, flies were also separated by sex, with male flies kept on the control diet and female flies on the supplemented diets. After hatching, male and female offspring (F1) were maintained on the control diet.

**FIGURE 4:**
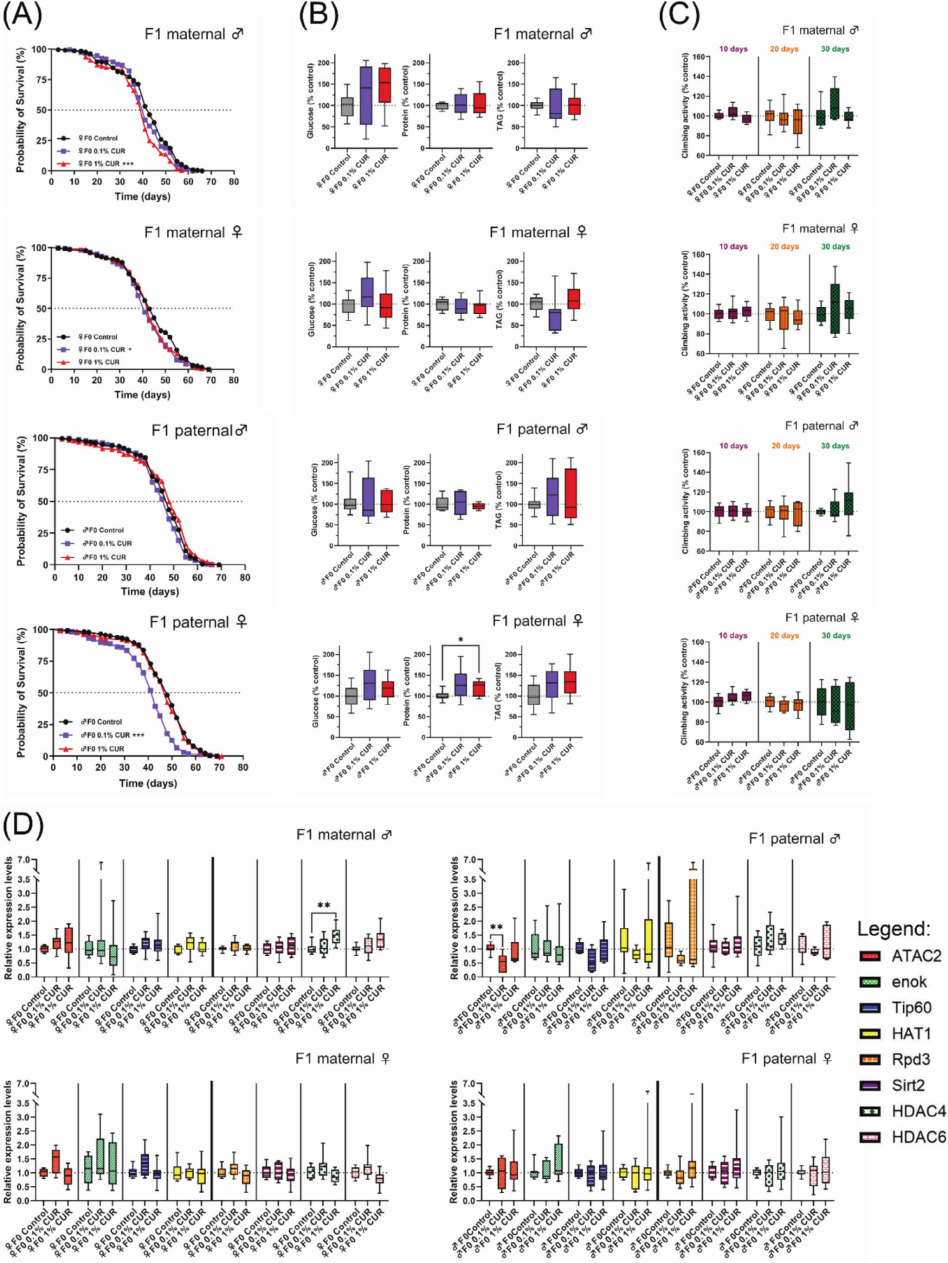
Parental feeding with curcumin influences lifespan, metabolism, and gene expression in F1 offspring. (A) Lifespan curves show a shorter median lifespan in the offspring of curcumin‐fed parents, but the effects are dependent on sex and curcumin concentration. (B) Glucose, protein, and TAG levels in F1 offspring were measured after 30 days on the control diet. Protein levels were higher in female offspring of mothers fed on 1% curcumin. (C) Climbing ability of F1 offspring was measured after 10, 20, and 30 days on the control diet and was not affected by parental curcumin feeding. (D) Expression levels of selected HAT and HDAC genes in the F1 offspring were measured after 30 days on the control diet. Only male offspring showed alterations in gene expression, caused by either maternal (♀F0, HDAC4 upregulation) or paternal (♂F0, ATAC2 downregulation) feeding with curcumin. ♂F0: paternal feeding; ♀F0: maternal feeding. The boxplots in panels (B–D) show median, minimum, and maximum values, and lifespan is shown as Kaplan–Meier curves. Three independent experiments, each with three replicates, were performed. Data represent three independent experiments, each with three replicates. (A) Log‐rank Test (B and D) ANOVA or Welch‐ANOVA or Kruskal–Wallis (C) Mixed model analysis (*p < 0.05; **p < 0.01; ***p < 0.001). CUR, curcumin.

The analysis of longevity in the F1 generation revealed a surprising sex‐dependent decrease in median lifespan. Offspring of curcumin‐fed mothers showed a notable decrease in median lifespan that was the same in both sexes (♂ 1% curcumin −4.7%, ♀ 0.1% curcumin −4.7%). However, male offspring of curcumin‐fed fathers showed no differences in lifespan compared to the control diet group, whereas female offspring of curcumin‐fed fathers (0.1% curcumin) had a median lifespan that was 5 days (−10.4%) shorter than that of the paternal control diet group (Figure 4A), as well as shorter mean and maximum lifespans (Table 4). The climbing assay was again used to assess fitness after 10, 20, and 30 days, but revealed no substantial differences in climbing activity between treated and untreated parental groups (Figure 4C). Changes in whole glucose, protein, and TAG levels were measured after 30 days of treatment. Glucose levels remained unchanged in response to parental curcumin‐supplemented diets, but protein levels increased in the female offspring of curcumin‐fed fathers (♂ F0 0.1% curcumin 29% *p* = 0.107 not significant.; ♂ F0 1% curcumin 20% *p* = 0.038). The observed increase in protein levels of 29% in female flies of the paternal 0.1% curcumin feeding is not statistically significant due to high standard deviations, but is of biological interest since only female offspring of mothers fed with curcumin show increased protein levels. Differences in protein levels were also observed between the male and female offspring of 1% curcumin‐fed fathers (*p* = 0.017), which further supports our theory of differential regulation of protein levels in male and female flies due to curcumin feeding.

**TABLE 4:** Median, mean, and maximum lifespan of F1 offspring from curcumin‐fed parents.

	Treatment	Median lifespan		Mean lifespan		Maximum lifespan	
Maternal	♂ Control	43 days	100%	100%		100%	
	♂ 0.1% CUR	41 days	95.3% n.s.	99% ± 18.9%	n.s.	97% ± 2.4%	n.s.
	♂ 1% CUR	41 days	95.3% ***	90% ± 3.8%	n.s.	89% ± 5.4%	n.s.
	♀ Control	43 days	100%	100%		100%	
	♀ 0.1% CUR	41 days	95.3 *	96% ± 14.5%	n.s.	101% ± 6.6%	n.s.
	♀ 1% CUR	43 days	100% n.s.	99% ± 6.9%	n.s.	100% ± 2.9%	n.s.
Paternal	♂ Control	48 days	100%	100%		100%	
	♂ 0.1% CUR	48 days	100% n.s.	98% ± 13.9%	n.s.	98% ± 5.7%	n.s.
	♂ 1% CUR	48 days	100% n.s.	101% ± 11.9%	n.s.	103% ± 5.9%	n.s.
	♀ Control	48 days	100%	100%		100%	
	♀ 0.1% CUR	43 days	89.6% ***	85% ± 4.0%	*	87% ± 1.7%	**
	♀ 1% CUR	48 days	100% n.s.	98% ± 7.8%	n.s.	98% ± 4.5%	n.s.

*Note:* n.s, not significant. Presented are median lifespan with log‐rank test and mean and maximum lifespan (last 10% of living flies) with ANOVA in mean ± SD, respectively. CUR, curcumin.

* *p* < 0.05.

** *p* < 0.01.

*** *p* < 0.001.

TAG levels were 24% lower in the female offspring of 0.1% curcumin‐fed mothers but 12% higher in the female offspring of 1% curcumin‐fed mothers, when compared to control diets (*p* = 0.071, not significant; Figure 4B). HAT and HDAC gene expression levels were measured in F1 flies after 30 days. Significant differences were only observed in male offspring, regardless of maternal and paternal curcumin treatment. Maternal treatment with 1% curcumin increased the expression levels of *HDAC4*, whereas paternal treatment with 0.1% curcumin reduced the expression levels of *ATAC2* significantly (*p* = 0.002) and *Tip60* non‐significantly (*p* = 0.068; Figure 4D).

## 4 Discussion

The anti‐inflammatory and anti‐oxidant properties of curcumin have been shown to increase the lifespan of model organisms, including nematodes ( *Caenorhabditis elegans* ), fruit flies, red flour beetles, and mice [24, 25, 26, 30]. The health benefits of curcumin involve diverse mechanisms of action, including depletion of reactive oxygen species, the promotion of superoxide dismutase and catalase activity [25, 44, 45], the inhibition of NF‐κB and the reduction of inflammatory markers [46]. Curcumin can also have adverse effects, particularly at high doses, but the underlying mechanisms are not fully disclosed [7, 31, 33, 34]. In fruit flies, acute toxicity tests showed that 1% curcumin in the diet had no detrimental effects, whereas higher concentrations of up to 10% inhibited oviposition, increased the duration of development, and led to a lower egg‐to‐adult survival ratio [34]. The concentration of curcumin applied to fruit flies in different studies has ranged from 5 μM to 270 mM [29, 34], and the time point and duration of application have also differed widely [47]. Furthermore, the effect of curcumin on fruit flies depends on their genetic background and sex [36]. Therefore, we applied lower (0.1%) and higher (1%) concentrations of curcumin, which have been shown to elicit a positive effect on lifespan in flies and should be sufficient to induce inheritable changes. These concentrations were also selected to counteract the low bioavailability of curcumin and to investigate its potential hormetic effect [32, 35, 48].

We found no evidence that curcumin suppressed food intake, indicating that the prolonged lifespan we observed was not caused by caloric restriction (Figure 1A). Indeed, female flies increased their food intake by 175%, which is consistent with previous reports [49]. The mechanism may be similar to that observed in flies treated with isoflavones, which were retained in the intestines of female flies in significantly greater quantities than in males, with males also expressing higher levels of the xenobiotic transporter gene *Mdr5* [37]. It remains unclear whether the increased food intake we observed is a persistent behavioral change or one that only occurs during curcumin treatment. This should be further addressed by looking for dose‐dependent effects in female flies on a curcumin‐supplemented diet.

Given that the body weight of both male and female flies remained constant, it was unclear where the additional energy from the increased food intake was utilized. To investigate this phenomenon, we analyzed the general activity of flies over a 10‐day period, surprisingly revealing a significant reduction in activity in both sexes following curcumin treatment (Figure 2B). Analysis of the excretion rate would have determined whether curcumin affects feeding behavior or if curcumin accumulates in the intestine. Curcumin affects genes involved in the regulation of dopamine synthesis, which is involved in the initiation of food consumption [49, 50], thus providing a mechanism for curcumin to influence feeding behavior. Male and female flies exhibited different activity patterns during the 24 h diurnal cycle (day, dusk, night, and dawn), highlighting the need to conduct experiments separately for male and female flies and to consider the timing of experiments.

Both concentrations of curcumin significantly prolonged the median lifespan of male and female flies, demonstrating the beneficial effect of our selected concentrations (Figure 1C). Curcumin at 0.1% also increased the maximum lifespan of male flies. We used a climbing assay to determine relative fitness levels and observed that the fitness of flies increased after 30 days of curcumin treatment, with male flies already exhibiting an increase after 20 days. Female flies showed a detrimental effect after 20 days feeding on curcumin but an increase after 30 days (Figure 2A). This finding is consistent with previous reports that curcumin improves locomotion in general [51], but particularly in fly models of diseases such as Alzheimer's disease or Huntington's disease [52, 53, 54]. However, the observed decline in the climbing activity of female flies after feeding for 20 days on 1% curcumin may indicate a potential toxic effect on locomotion.

Curcumin triggered changes in the metabolic composition of flies (protein, glucose and TAG level) that partially persisted to the F1 generation and depended on sex and curcumin concentration, which ultimately led to either beneficial or detrimental effects (Figure 3A,B and Figure 4B). Female flies had higher protein levels after 8 days on a curcumin‐supplemented diet, and this is associated with an increased lifespan and also has affected development and metabolism in offspring [55, 56]. After 30 days, the protein levels in the female flies tended to remain elevated compared to the control group, but this difference was not statistically significant (Figure 3B). The offspring of curcumin‐fed mothers had a significantly shorter lifespan, despite the elevated protein levels in mothers at the time of egg laying, indicating that this metabolic effect is not inherited (Figure 4A,B). Lower TAG levels were observed in the female offspring of curcumin‐fed mothers than in the control group, although this difference was not statistically significant due to high standard deviations (Figure 4B). This finding might be consistent with the idea that reduced TAG levels are associated with a shorter lifespan in flies [57, 58] and this association has also been observed in response to curcumin [29]. Another supporting factor is the slightly higher TAG levels (though not statistically significant) in the female offspring of males fed on curcumin, which further implicates the sex‐specific effects of curcumin feeding.

In further support of our findings, the transcriptomic and metabolomic analysis of flies fed on 1% curcumin for 5 days revealed a downregulation of genes involved in lipid metabolic processes with triglyceride lipase activity (CG11608, CG18530, and CG6753) and different expression levels for genes involved in the regulation of feeding behavior, including a downregulation of *Or59a* [49]. Our gene expression analysis revealed a similar, complex impact in parental flies, suggesting that the changes in gene expression and metabolism may not be controlled in the same manner in both sexes, but rather through different mechanisms (Figure 4D). Feeding male flies with curcumin for 8 days inhibited the expression of *enok*, which has a role in processes such as cell growth and proliferation, and the regulation of transposon activity [59, 60]. The loss of *enok* expression could increase transposon activity, potentially leading to genomic instability resulting in mutations and cancer [61]. *HAT1* expression increased in curcumin‐fed male flies after 30 days. HAT1 has a comparable function to Enok [62], and its higher expression levels could stabilize the genome later in older flies by limiting transposon activity. Feeding female flies with curcumin for 8 days inhibited the expression of *Tip60*, *HAT1*, *Rpd3*, and *Sirt2*, but these expression profiles normalized after 30 days. Lower *Rpd3* and higher *Sirt2* expression are associated with a longer lifespan [63, 64, 65]. However, we observed lower levels of *Rpd3* and *Sirt2* mRNA in female flies after 8 days of curcumin treatment. This may have contributed to the reduced lifespan of the F1 offspring because this phenomenon has also been observed in mutants with lower *Rpd3* and *Sirt2* expression [65]. Furthermore, we observed lower expression levels of *Tip60*, a gene implicated in cell cycle regulation and known to be affected by curcumin [66, 67]. *Tip60* is also involved in the regulation of the sleep–wake cycle in flies [68], which may explain the lower activity levels of flies in response to curcumin.

The changes in gene expression and metabolism contribute to a “detrimental status” in female flies with regard to the transgenerational impact of curcumin. This status changes with aging and later exerts a positive influence. These findings demonstrate that the impact of curcumin varies significantly according to age, sex, and dose [35]. A clear distinction between maternal and paternal curcumin feeding in both sexes of offspring is evident, with maternal (but not paternal) feeding significantly increasing the expression of *HDAC4* in male offspring. Both *HDAC4* and *HDAC6* are known to influence lifespan by regulating multiple genes [20]. The male offspring of mothers fed on 1% curcumin had a shorter lifespan, which may reflect the stronger expression of *HDAC4* and *HDAC6*, although the increase was not statistically significant. Furthermore, the female offspring of curcumin‐fed fathers experienced the most significant decrease in median, mean, and maximum lifespan, whereas there was no change in male offspring. ATAC2, the human ortholog of CRBP2, is involved in the formation of the female germline and the regulation of intestinal stem cells (ISCs). *ATAC2* downregulation increases the number of ISCs and the lethality of mutants lacking the ATAC complex, in which ATAC2 plays an important role, during larval stages [69, 70, 71]. We observed the downregulation of *ATAC2* in the male offspring of curcumin‐fed fathers but no change in female offspring, although only the female flies showed a reduced lifespan. This suggests curcumin acts differently in male and female flies and needs to be investigated in more detail. Climbing activity, which is influenced by curcumin in the F0 generation, was no longer affected in the F1 generation, suggesting that this aspect of curcumin activity is not inherited.

We have demonstrated that curcumin has a transgenerational and sex‐dependent detrimental effect. Curcumin‐fed fathers and mothers influence the F1 in different ways. Young female flies showed a greater response to curcumin than age‐matched males, which underscores the significance of incorporating sex and age in the analysis of curcumin's effects. A detailed analysis of specific epigenetic changes, such as histone acetylation patterns (e.g., H3K9ac or H4K16ac) in both generations, would be advantageous to get a deeper insight into the underlying epigenetic mechanisms. To unravel the potential mechanistic link in our study, mutants of single or combined HATs and HDACs, like knockdown or overexpression, would help to clarify the epigenetic impact of curcumin in depth. Importantly, the integrity of the gut plays a critical role in longevity [72] and also displays significant sex‐specific differences, including a larger gut in females [73] and fewer proliferating ISCs in males [74, 75], which may partly explain the sex‐dependent effects of dietary curcumin. Further research on the sex‐specific and transgenerational effects of curcumin, including the assessment of the gut integrity by e.g., the SMURF‐Assay [72], is required, also considering further generations after the current F1 generation. This is because curcumin is widely used by humans as a health‐promoting dietary supplement, and it acts on multiple pathways that can influence a number of diseases, including cancers.

## Conflicts of Interest

The authors declare no conflicts of interest.

## Supporting information


**Figure S1:** Sectioning of vials for determination of climbing score.
Section numbers: 1: all flies on the bottom; 2: all flies on the wall and below the first line; 3: all flies above the first line and below the second line; 4: all flies above the second line.
**Figure S2:** Body weight of CUR‐fed flies did not show significant differences after 30 days of feeding with 0.1% or 1% curcumin compared to the control diet. Three independent experiments, each with three replicates, were performed. Boxplots show median, minimum and maximum values. Data represent three independent experiments, each with three replicates (A) Kruskal–Wallis (B) One‐Way ANOVA (*p > 0.05; **p < 0.01; ***p < 0.001).

## Data Availability

The data that support the findings of this study are available from the corresponding author upon reasonable request.
