# Supplementary material for: Curcumin Induces Transgenerational and Sex‐Specific Effects on Lifespan, Gene Expression, and Metabolism in the Fruit Fly Drosophila melanogaster
Source: Biofactors. 2025 Aug 1;51(4):e70039. doi: 10.1002/biof.70039 (PMC12317048; doi:10.1002/biof.70039)

Supplementary information


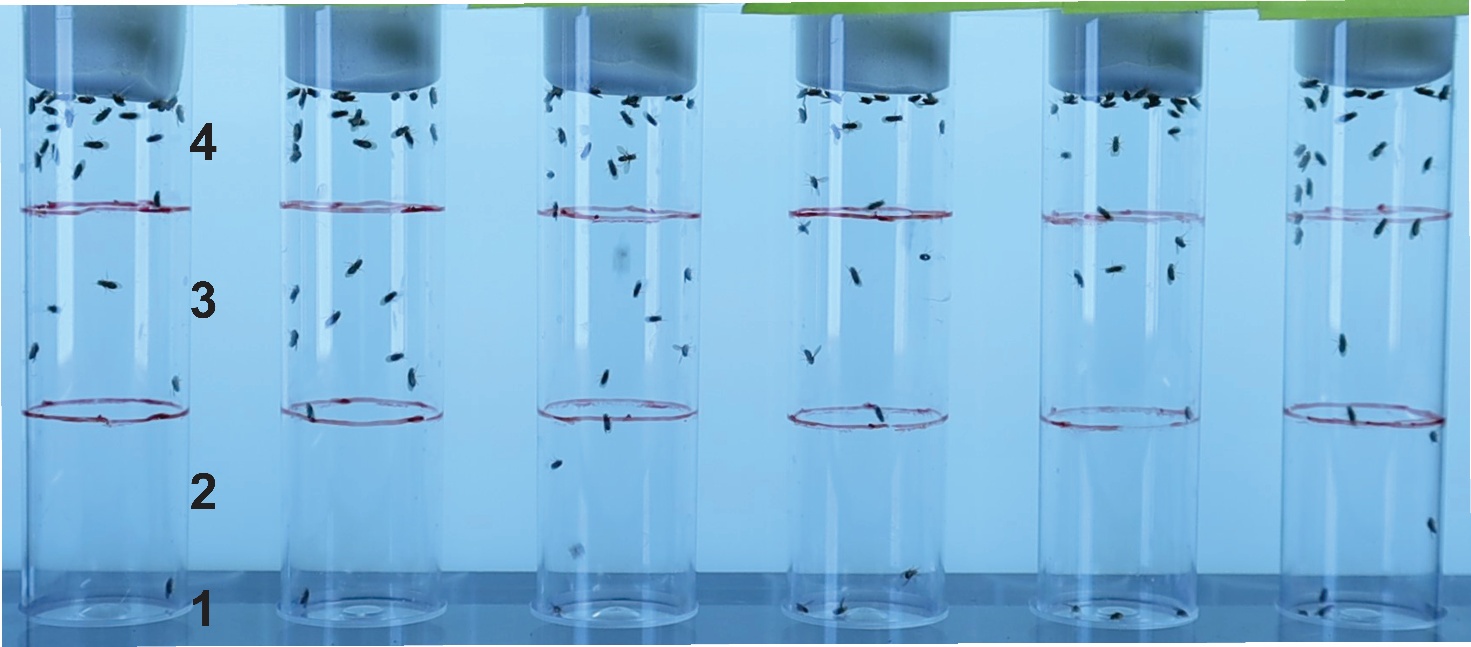


Figure S1: Sectioning of vials for determination of climbing score.

Section numbers: 1: all flies on the bottom; 2: all flies on the wall and below the first line; 3: all flies above the first line and below the second line; 4: all flies above the second line.

Figure S2: Body weight of CUR-fed flies did not show significant differences after 30 days of feeding with 0.1% or 1% curcumin compared to the control diet. Three independent experiments, each with three replicates, were performed. Boxplots show median, minimum and maximum values. Data represent three independent experiments, each with three replicates (A) Kruskal-Wallis (B) One-Way ANOVA (*p > 0.05; **p < 0.01; ***p < 0.001).


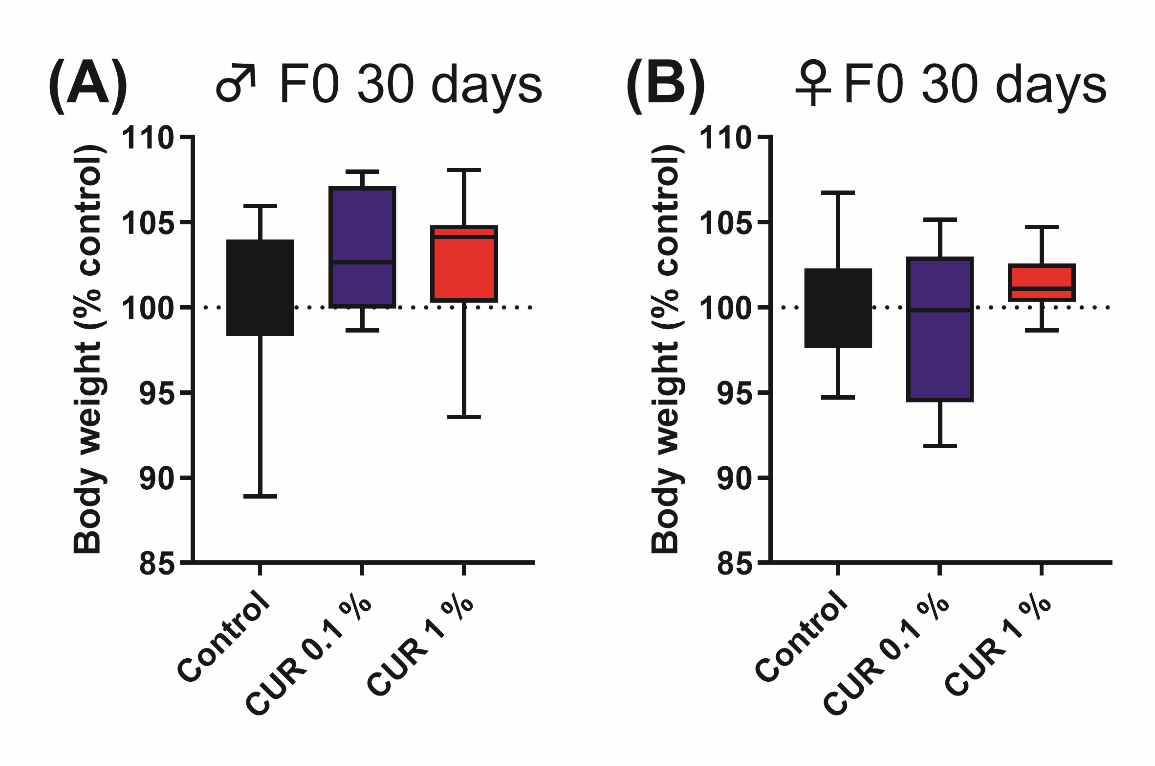

Supplement: Supplementary file 1 — Figure S1: Sectioning of vials for determination of climbing score. Section numbers: 1: all flies on the bottom; 2: all flies on the wall and below the first line; 3: all flies above the first line and below the second line; 4: all flies above the second line. Figure S2: Body weight of CUR‐fed flies did not show significant differences after 30 days of feeding with 0.1% or 1% curcumin compared to the control diet. Three independent experiments, each with three replicates, were performed. Boxplots show median, minimum and maximum values. Data represent three independent experiments, each with three replicates (A) Kruskal–Wallis (B) One‐Way ANOVA (*p > 0.05; **p < 0.01; ***p < 0.001). [file BIOF-51-0-s001.docx]
